# Supplementary material for: Netcombin: An algorithm for constructing optimal phylogenetic network from rooted triplets
Source: PLoS One. 2020 Sep 18;15(9):e0227842. doi: 10.1371/journal.pone.0227842 (PMC7500971; doi:10.1371/journal.pone.0227842)
Supplement: S4 File — (ZIP) [file pone.0227842.s004.zip › SequenceData-Triplet/sequenceData-10-20-30-0-Triplet/length 300, numseq 40, rate 10^-7/2 seq.pdf]

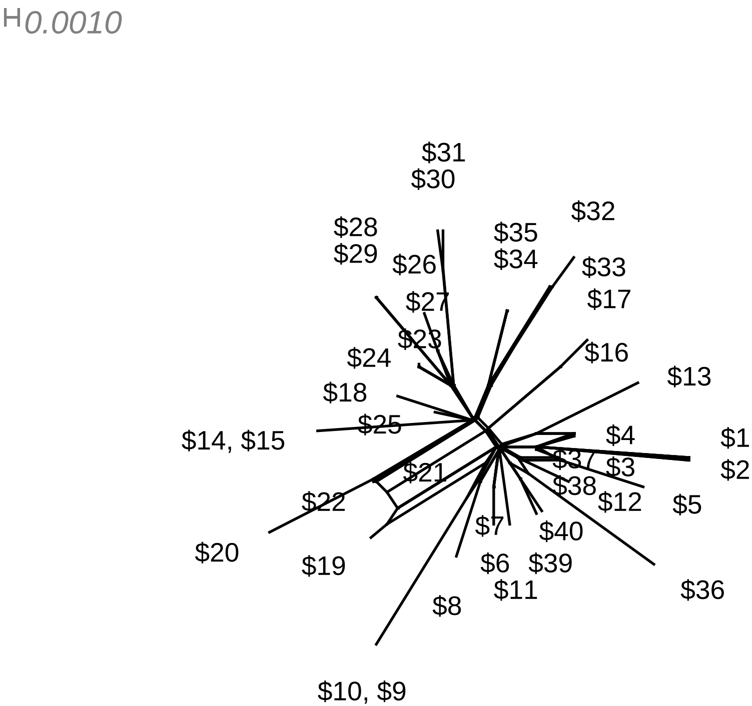
0.0010
$31
$30
$32
$28
$35
$29
$34
$26
$33
$17
$27
$23
$24
$16
$13
$18
$25
$14, $15
$4
$1
$37
$3
$2
$21
$38
$22
$12
$5
$7
$40
$20
$19
$6
$39
$11
$36
$8
$10, $9
